# Amplitudes of Pain-Related Evoked Potentials Are Useful to Detect Small Fiber Involvement in Painful Mixed Fiber Neuropathies in Addition to Quantitative Sensory Testing – An Electrophysiological Study

**DOI:** 10.3389/fneur.2015.00244

**Published:** 2015-12-07

**Authors:** Niels Hansen, Ann-Kathrin Kahn, Daniel Zeller, Zaza Katsarava, Claudia Sommer, Nurcan Üçeyler

**Affiliations:** ^1^Department of Neurology, University of Würzburg, Würzburg, Germany; ^2^Department of Neurophysiology, Ruhr-University Bochum, Bochum, Germany; ^3^Department of Epileptology, University of Bonn, Bonn, Germany; ^4^Department of Neurology, Evangelisches Krankenhaus, Unna, Germany

**Keywords:** mixed fiber neuropathy, pain-related evoked potentials, Aδ- and C-fibers, neuropathic pain, burning pain, quantitative sensory testing

## Abstract

To investigate the usefulness of pain-related evoked potentials (PREP) elicited by electrical stimulation for the identification of small fiber involvement in patients with mixed fiber neuropathy (MFN). Eleven MFN patients with clinical signs of large fiber impairment and neuropathic pain and ten healthy controls underwent clinical and electrophysiological evaluation. Small fiber function, electrical conductivity and morphology were examined by quantitative sensory testing (QST), PREP, and skin punch biopsy. MFN was diagnosed following clinical and electrophysiological examination (chronic inflammatory demyelinating neuropathy: *n* = 6; vasculitic neuropathy: *n* = 3; chronic axonal ­neuropathy: *n* = 2). The majority of patients with MFN characterized their pain by descriptors that mainly represent C-fiber-mediated pain. In QST, patients displayed elevated cold, warm, mechanical, and vibration detection thresholds and cold pain thresholds indicative of MFN. PREP amplitudes in patients correlated with cold (*p* < 0.05) and warm detection thresholds (*p* < 0.05). Burning pain and the presence of par-/dysesthesias correlated negatively with PREP amplitudes (*p* < 0.05). PREP amplitudes correlating with cold and warm detection thresholds, burning pain, and par-/dysesthesias support employing PREP amplitudes as an additional tool in conjunction with QST for detecting small fiber impairment in patients with MFN.

## Introduction

Painful polyneuropathies of different origin often affect large- and small-nerve fibers (1, 2) and are therefore termed mixed fiber neuropathies (MFN). Evaluating small fiber impairment can facilitate the differential diagnosis of painful MFN. Familial amyloid polyneuropathy is an example of MFN that may initially be asymptomatic (3) and where assessment of small fiber function may allow an early diagnosis. Small fiber tests could be appropriate in clinical course and analyzing therapy efficacy (4). The latter is of particular importance, since efficacious pharmacotherapy is disposable for several acquired neuropathies (5). Small thinly myelinated as well as unmyelinated nerve fibers (Aδ- and C-fibers) can be assessed via neurological examination and quantitative sensory testing (QST) (6). However, both methods are subjective and very dependent on patients’ cooperation. Since standard nerve conduction studies fail to measure the impairment of small-caliber nerve fibers, alternative objective means are needed. Nociceptive laser-evoked potentials (LEP) and contact heat-evoked potentials (CHEP) are suitable electrophysiological methods to detect functional impairment of small fibers in painful MFN (7). However, LEP and CHEP have drawbacks in clinical routine such as expensive equipment and complex procedures (8). The recording of electrically elicited pain-related evoked potentials (PREP) via concentric electrodes (9) is a useful, non-invasive, and handy method to detect small fiber impairment in early diabetes (10), human immunodeficiency virus (HIV) associated neuropathy (11), Fabry disease (12), and fibromyalgia syndrome (13). Neuropathic pain, pathological sural nerve conduction, and pathological PREP have been obtained in patients with hepatitis C- and HIV infection-associated sensory neuropathies (11, 14). PREP are thus suitable for detecting small fiber involvement in a variety of conditions characterized by small fiber pathology.

Painful MFN is a neuropathy encountered frequently in clinical routine, for example in patients with chronic inflammatory demyelinating neuropathy (CIDP). CIDP patients often have small fiber impairment in addition to large fiber affection (15). Neuropathic pain in large fiber neuropathy patients indicates but does not ascertain the involvement of small nerve fibers (16, 17). To accelerate diagnosis and treatment, and to monitor the time course of small nerve fiber degeneration or pharmacotherapeutic efficacy in MFN patients, a simple and objective tool to determine small fiber involvement is required.

We therefore investigated the usefulness of PREP in assessing small fiber involvement in painful MFN. We additionally utilized QST as a standard instrument to retrieve small fiber function in neuropathy patients and compared PREP and QST data in MFN. We hypothesized that PREP amplitudes would be reduced and PREP latencies delayed particularly when elicited from the feet in patients with additional small fiber affection.

## Background

### Patients and Subjects

In this study, eleven patients with painful MFN (median age = 61 years, range: 37–83 years, three females and eight males) were prospectively enrolled in parallel to the recruitment of healthy control subjects. The diagnosis was made when clinical signs of large fiber affection as well as neuropathic pain coincided. The recruitment of patients was performed between 2009 and 2012 at the Department of Neurology, University of Würzburg. Inclusion criteria consist of female and male patients at the age of ≥18 years, confirmed diagnosis of painful motor or sensory polyneuropathy (by medical report, neurological examination, standard neurophysiology). Exclusion criteria were: pain of other than neuropathic origin (e.g., dermatologic, orthopedic) or abnormal blood investigations not related to the neuropathy. A robust and reproducible PREP response was a prerequisite for including MFN patients in our study, as we were dedicated to delineating the differences between PREP latencies and amplitudes in patients and controls.

We compared our data with those from ten healthy age- and gender-matched controls recruited during our study (median age = 55 years, range: 26–67 years; four females and six males). Inclusion criteria for control subjects comprised the following items: ≥18 years, unremarkable sural nerve conduction, no neuropathy and no report of pain (neuropathic pain or other causes of pain). The age and gender did not vary significantly between patients and control subjects (*p* > 0.05 each). The performance of the study was in agreement with the Declaration of Helsinki. The study was authorized by the Würzburg Medical Faculty Ethics Committee. Recorded informed consent was received from all participants of the study.

### Neurological Investigation, Pain and Depression Questionnaires, Neuropathy Scales

Every patient was subject to a neurological examination and was investigated by employing the German translations of pain and depression questionnaires as well as neuropathy scales. Severity of neuropathy was estimated using a variation of the Neuropathy Deficit Score (NDS) and the Neuropathy Symptoms Score (NSS) (18, 19). The Neuropathic Pain Symptom Inventory (NPSI; 24-h recall) (20, 21) assesses neuropathic pain characteristics and intensity; the resulting sum score goes from 0 (no pain) to 1 (maximum pain). The McGill pain questionnaire (22) was employed to characterize patients’ pain by distinct affective and sensory descriptors. Patients’ answers to each of the 78 questions ranged between 0 (inappropriate) and 4 (fully appropriate). To discriminate between Aδ- and C-fiber excitation attributed pain, a rating test utilizing the adjectives “dull,” “pressing” and “pricking” from the McGill pain questionnaire was applied according to Beissner (23). Pain was attributed to C-fiber excitation if the sum of selecting “dull” or “pressing” was higher than that of choosing “pricking” (23). Pain magnitude (score based on three pain intensity items) and impact of pain (score of three items on pain perturbation with everyday life activities) was investigated by the Graded Chronic Pain Scale (GCPS; an altered version consisting of a 4-week recall) (24).

To additionally identify potential depressive symptoms, we utilized the Center for Epidemiologic Studies Depression Scale (“Allgemeine Depressionsskala,” ADS; 1-week recall) (25). The maximum ADS score is amounted to 60; a total score ≥ 16 is expected to be relevant in clinical practice.

To diagnose CIDP the Inflammatory Neuropathy Cause and Treatment (INCAT) criteria (26) were applied; the EFNS/PNS criteria were used to diagnose non-systemic vasculitic neuropathy (27).

### Quantitative Sensory Testing

All patients and healthy controls underwent QST. Following current recommendations (28–31), we log-transformed the QST parameters and calculated *z*-scores, which allows normalization of the patient and control data to the data of the controls (mean and SD), i.e., also the data of the ten controls were referenced to themselves.

#### Parameters

We performed QST in each study participant on the dorsum of the foot following the standardized protocol of the German Research Network Neuropathic Pain (Deutscher Forschungsverbund Neuropathischer Schmerz, DFNS) and using a calibrated device (Somedic, Hörby, Sweden) (28, 29). We assessed the successive determinants: cold detection threshold (CDT), cold pain threshold (CPT), warm detection threshold (WDT), heat pain threshold (HPT), capability to identify temperature alterations (thermal sensory limen, TSL), mechanical detection threshold (MDT), mechanical pain sensitivity (MPS), mechanical pain threshold (MPT), pressure pain threshold (PPT), and vibration detection threshold (VDT).

In brief: for CDT and HDT, a thermode was positioned above the dermis and the temperature lowered or raised starting at 32°C (min. 10°C, max. 50°C). Each control subject or patient pushed a knob when the temperature he or she sensed was cold or warm, respectively. CPT and HPT were assessed accordingly. TSL was determined by alternately changing the thermode’s temperature beginning at 32°C; the subject pushed a button to indicate when a change in temperature was felt. MDT was determined by stimulating the skin with calibrated von Frey filaments; the examination started with a von Frey filament of 16 mN and the intensity reduced or increased in the filament being sensed by the subject defining the MDT (range 0.25–512 mN). Calibrated pinpricks (range 0.25–512 mN) were applied to determine the MPT; to determine the MPS, the subject was stimulated with a pin-prick and thereafter with a brush, a Q-tip or a cotton ball; the subject then rated the degree of strength of the pinprick stimulus on a chart from 0 to 100. The VDT was determined using a tuning fork. A calibrated algesiometer (Wagner Instruments, USA) was used to assess the PPT.

#### Assessment

For group analysis, patients’ data were compared with data from 10 healthy control subjects following the same protocol at our department. According to current recommendations (28–31), QST raw values were log-transformed to reach normal distribution (CDT, MDT, MPS, PPT, TSL, WDT). The CPT, HPT, and VDT parameters were not log-transformed (28, 29), as the ratio for raw data to log-transformed data did not cross a factor of three, and as one prerequisite for log-transformation (i.e., determination of the exact zero-threshold of the respective parameter) was unfulfilled (29). With these data, we calculated a *z*-score utilizing the formula: *z*-score [(value of the patient - mean value of control subjects)/SD of control subjects]. Calculating the *z*-score allows the normalization of patient data to the control group. Positive *z*-scores reveal a gain of function whereas negative *z*-scores point to a loss of function in comparison to the controls. Since in this formula the controls’ QST values are normalized to their own mean and SD, the controls’ QST *z*-score values result in zero with a SD of one. Since the MPS examination may result in “zero,” we applied the Bartlett procedure (31, 32) and added a constant of 0.1 to the MPS results for statistical reasons (33). *Z*-values below or above ±1.96 were determined as aberrant (30, 34).

For individual assessments of QST results, we used published normative data (30). The controls’ QST values were within the extent of released QST data from healthy control groups (12, 13, 35, 36).

### Electrophysiological Assessment

All patients and healthy controls underwent nerve conduction studies. The right sural and tibial nerves in patients and the right sural nerves in healthy controls were examined utilizing electrodes according to standard procedures (37) to determine large fiber polyneuropathy. Findings were compared with our laboratory normal values for adults. Our laboratory normal values consist of an antidromic sural nerve action potential (SNAP) amplitude ≥10 μV for age <65 years and ≥5 μV for age >65 years. The normal values for the sural nerve conduction velocity (NCV) were >40 m/s for all age groups. The tibial nerve compound motor action potential (CMAP) was composed of values ≥10 mV and the tibial NCV ≥40 m/s for all age groups.

### Pain-Related Evoked Potentials

All patients and healthy controls underwent PREP derived from Cz via a needle electrode sited subcutaneously and referenced to connected ear lobes (A1-A2, international 10–20 system) as formerly mentioned in detail (13). Signal Software (Version 2-16, Cambridge Electronic Design, Lt., Cambridge, UK) was used. The potentials were elicited via electrical stimulation with a constant current stimulator (DS7A, Welwyn Garden City, UK) at the right and left foot (dorsum) employing planar concentric electrodes attached to the skin (Inomed Medizintechnik GmbH, Lübeck, Germany). This stimulation paradigm consists of 20 triple pulses at twofold intensity of each pain threshold lasting 0.5 ms, a triple pulse interval of 5 ms and random inter-stimulus interval of 15–17 s. The recording set-up comprises the following adjustments: gain: ×5000, bandwidth: 1 Hz–1 kHz, digitalization sampling rate: 2.5 kHz and sweep length: 400 ms. Single pain threshold was ascertained by stimulating twice with current intensities that rose and fell until the person remarked a pin-prick perception. The MATLAB software (Version 7.7.0471, Ismaning, Germany) was utilized to survey averaged curves (*n* = 20 single sweeps) for reproducible N1- (i.e., first negative peak), P1- (i.e., subsequent positive peak) latencies, and peak-to-peak amplitudes (PPA). Every curve was analyzed off-line via an examiner blinded to the diagnosis on coded data files. Recordings containing technical or biological artifacts were excluded from the analysis. Only those recordings were included that showed robust and reproducible potentials. Additional exclusion criteria for PREP recordings were: history of epilepsy, cardiac pacemakers, and deep brain stimulators.

### Skin Biopsies

All patients underwent skin punch biopsies with a size of 5 mm (punch device: Stiefel, Offenbach, Germany) obtained to assess intra-epidermal nerve fiber density (IENFD). The locus for the removal of the biopsies was the lower extremity 10 cm proximal to the lateral malleolus. The skin probes were treated as specified formerly (38). Immunoreactions of the individual sections were caused by antibodies to protein-gene product (PGP) 9.5 (Ultraclone limited, Isle of Wight, UK, primary antibody; 1:800). The PGP 9.5 antibodies were combined with goat anti-rabbit IgG marked with cyanine 3.18 fluorescent sample (Amersham, USA, Cy3, secondary antibody; 1:100) (12). The IENFD was determined by an investigator blinded to the individual sample’s identification according to released approaches (39).

### Statistical Analysis

We used Statistica 64 10 (Copyright 1984–2011, Tulsa, OK, USA) for statistical analysis and Sigma Plot 11.0 for Windows (Copyright 2008, San Jose, California, USA) for graph creation. Normally distributed data was subjected to the Shapiro–Wilk test. To compare data without normal distribution, we applied the non-parametric Mann–Whitney *U* test, and the parametric student’s *t*-test to compare data with normal distribution. Data without normal distribution are shown as median and range (demographic data, questionnaires, sural nerve electrophysiology data), whereas data that show a normal distribution are illustrated as mean ± SD (PREP as well as QST data). For comparison with previously published data, the McGill pain questionnaire results are depicted as mean ± SD. For correlation analysis, the Pearson Product Moment correlation was utilized. The level of significance was set to *p* < 0.05.

## Results

### Questionnaire Results, Clinical Findings, and Standard Nerve Conduction Results

Our study cohort’s demographics, neuropathy scale scores, questionnaire results, and electrophysiology data are summarized in Table 1. Diagnoses and characteristics documented during the neurological examination are listed in Table S1 in Supplementary Material. Eleven patients with neuropathic pain (i.e., pain of ≥4/10 on a numeric rating scale, NRS) and signs of large-fiber involvement were classified as patients with painful MFN. The controls comprised ten healthy subjects from our department not presenting any neuropathy, neuropathic pain, or other causes of pain. The patient group consisted of six patients with CIDP (26), three patients with vasculitic neuropathy (27), and two patients with chronic axonal neuropathies. Neurological examination of the lower limb revealed pathological findings in all patients, whereas the healthy controls revealed no clinical abnormalities: 11/11 (100%) patients reported hypoesthesia to touch, 6/11 (54%) patients had thermal hypoesthesia, 11/11 (100%) patients had pallhypoesthesia, 7/11 (64%) patients presented pareses, and 10/11 (91%) patients had hyporeflexia. Autonomic dysfunction was present in 3/11 (27%) patients. One to two patients each (9–18%) reported disturbance in micturition or nycturia, cardiac arrhythmia, diarrhea, erectile dysfunction, and esophageal dysmotility. Nerve conduction studies of the tibial nerve revealed reduced CMAP in 10/11 (91%) patients and slowed NCV in 9/11 (82%) patients. Either the CMAP or NCV was abnormal in all patients. Electrophysiological assessment of the sural nerve (Table 1) showed reduced SNAP in 4/6 (67%) and no SNAP in 1/6 (17%) patients aged <65 years and reduced SNAP in 4/5 (80%) and no SNAP in 1/5 (20%) of patients aged >65 years. NCV of the sural nerve was abnormal in 4/11 (36%) patients. In contrast, the control subjects’ sural nerve conduction examinations were normal. 7/11 (64%) of patients received continious analgesic treatment (pregabalin *n* = 5, gabapentin *n* = 2), whereas the controls received no analgesic therapy.

**Table 1:** **Demographic, questionnaire, and neurophysiological data of patients with mixed fiber neuropathy and controls**.

	MFN patients	Controls	*p*-value
**Demographic data**			
Number (male, female)	11 (8, 3)	10 (6, 4)	ns
Median age years (range)	61 (37–83)	55 (26–67)	ns
Median disease duration in years (range)	2 (0.1–4.5)		
**Questionnaires**			
Current pain intensity (NRS; 0–10)	3 (0–7)	0	*p* < 0.05
Median NPSI sum score	0.23 (0.0–0.65)	0	*p* < 0.05
Median GCPS sum score (intensity)	53.3 (37–73)	0	*p* < 0.05
Median GCPS sum score (impairment)	1.5 (0–5)	0	*p* < 0.05
Median ADS score	26 (0–37)	0	*p* < 0.05
Median NDS sum score (0–272)	42 (20–67)	0	*p* < 0.05
Median NSS sum score (0–18)	5 (0–12)	0	*p* < 0.05
**Electrophysiology**			
**Sural nerve**			
Sensory nerve action potential (μV)	4.7 (0–17.1)	21.2 (9–37.2)	*p* < 0.05
Nerve conduction velocity (m/s)	41 (0–46.1)	52.9 (44–56.5)	ns

*ADS, Allgemeine Depressionsskala; GCPS, Graded Chronic Pain Scale; MFN, mixed fiber neuropathy; NDS, Neuropathy Disability Scale; NPSI, Neuropathic Pain Symptom Inventory; ns, not significant; NSS, Neuropathy Symptom Scale; NRS, Numerical Rating Scale*.

### QST Revealed Large and Small Fiber Impairment

**Figure 1:**
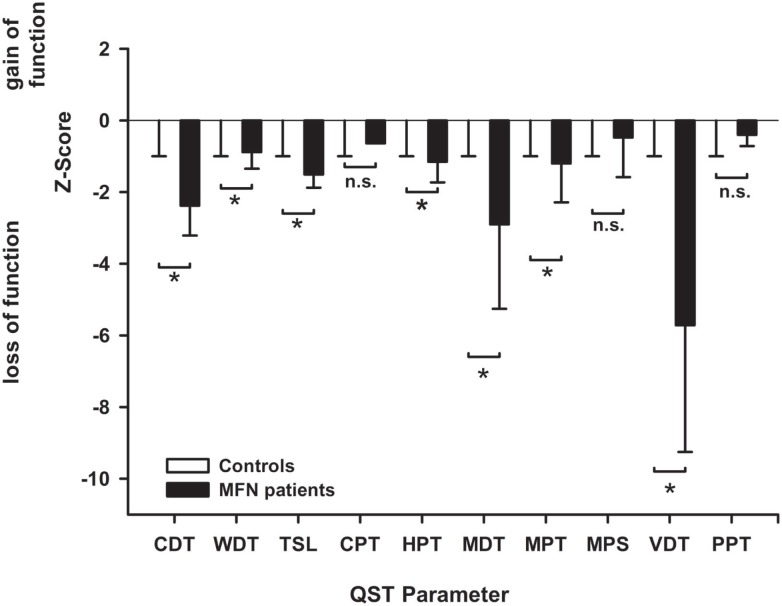
**Sensory profile of patients with mixed fiber neuropathy (MFN) and controls obtained by quantitative sensory testing (QST)**. The bar graphs depict the *z*-scores at the dorsal foot in MFN patients contrasted to healthy control subjects. Healthy controls are shown by white bar graphs, whereas MFN patients are represented by black bar graphs. The *z*-scores of healthy controls are zero with a SD of one due to the calculation of *z*-score (see methods). This is the reason why the white bar graphs are not seen and disappear in the zero-line. *z*-scores < 0 indicate a loss of function, *z*-scores > 0 exhibits a gain of function. MFN displays impaired cold (CDT), warm (WDT), mechanical (MDT), and vibration detection thresholds (VDT) as well as cold pain thresholds (CPT) compared to controls (*p* < 0.05) indicating deafferentation of small and large fibers. NB: Patients’ CPT was 10°C which is the cut-off value for CPT assessment during QST. Therefore, the CPT value does not have an error bar. Abbreviations: CPT, cold pain threshold; HPT, heat pain threshold; ability to detect temperature changes (thermal sensory limen, TSL); MPT, mechanical pain threshold; MPS, mechanical pain sensitivity; PPT, pressure pain threshold (PPT); VDT, vibration detection threshold. Data are expressed as mean and SD.*p < 0.05.

The detection thresholds of MFN patients as well as control subjects are illustrated in Table S2 in Supplementary Material. MFN patients had more elevated CDT and WDT than the control subjects (*p* < 0.05, Figure 1) translating to reduced cold and warm detection capacities and indicating functionally impaired Aδ- and C-fibers. MFN patients thus displayed warm and cold hypoesthesia compared to the controls. Furthermore, patients’ MDT and VDT were higher than those of controls (*p* < 0.05, Figure 1). These alterations suggest impaired Aß-fiber function and can be termed mechanical hypoesthesia compared to the controls. The increased HPT in MFN patients (*p* < 0.05 vs. controls, Figure 1) is compatible with impaired small fiber dysfunction and is called heat hypoalgesia. In healthy control subjects, the QST values fell within the confidence interval (± 1.96), indicating normal values.

### Altered Thermal Perception in Patients with MFN Correlates with PREP Amplitudes

**Figure 2:**
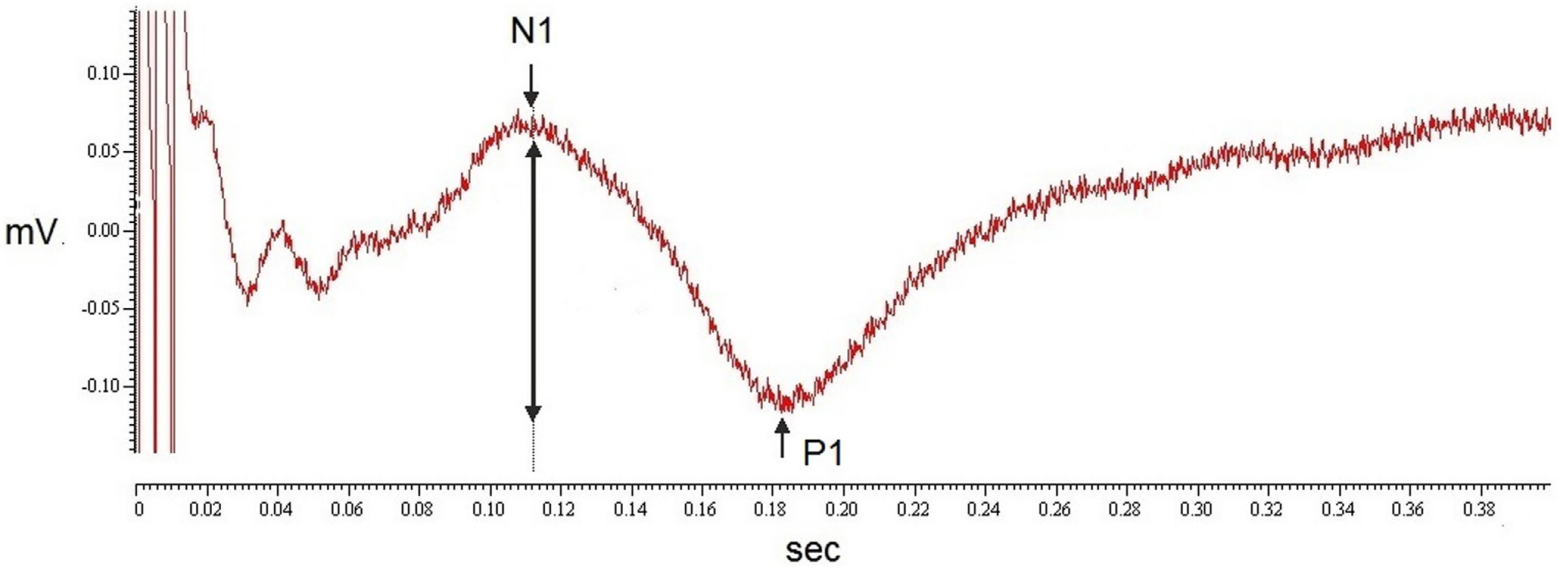
**A representative trace of a PREP recording in a control subject**. The figure shows an example of a representative PREP curve from a healthy control subject. The N1 and P1 latency are indicated by arrows; the peak-to-peak amplitude is indicated by a two-sided arrow.

**Figure 3:**
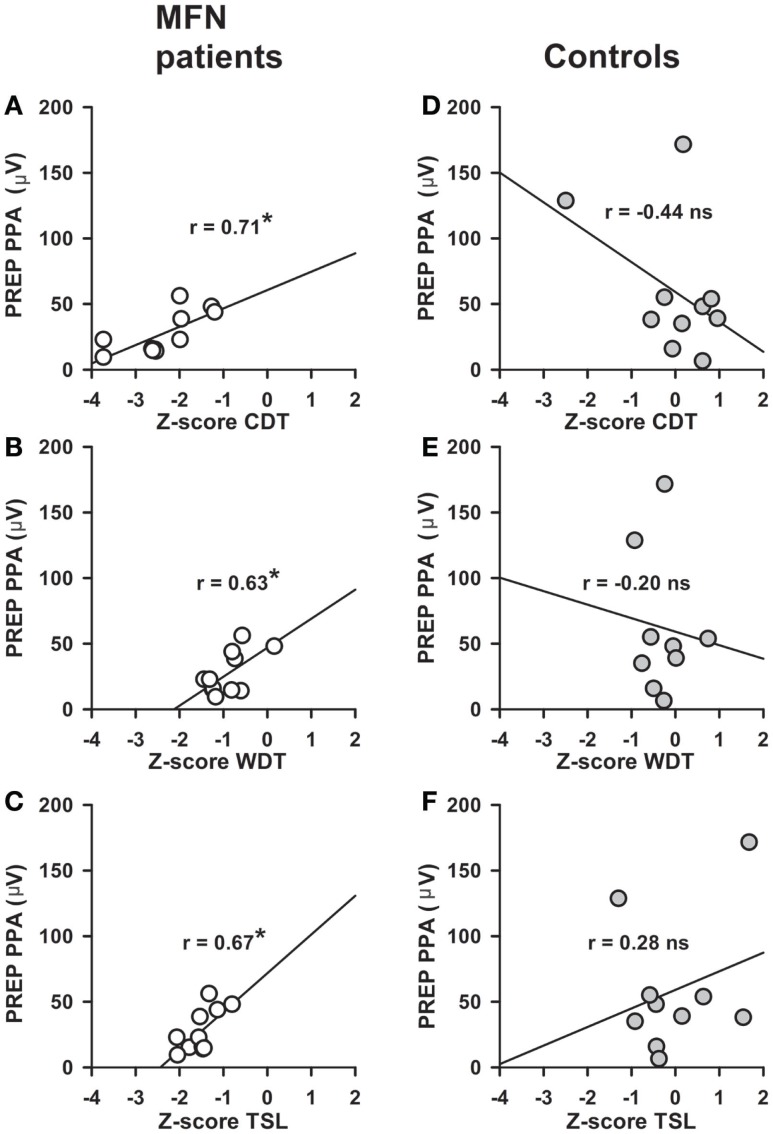
**Correlations between pain-related evoked potential (PREP) amplitudes and thermal perception in patients with mixed fiber neuropathy (MFN) and controls**. Correlations between PREP peak-to-peak amplitudes (PPA) recorded after electrical stimulation at the feet and quantitative sensory testing (QST) parameters are shown (cold detection threshold, CDT; warm detection threshold, WDT; thermal sensory limen, TSL). PREP peak-to-peak amplitude (PPA) correlated positively with CDT *z*-scores [*r* = 0.71, **(A)**], WDT *z*-scores [*r* = 0.63, **(B)**] and TSL *z*-scores [*r* = 0.67, **(C)**] in MFN patients **(A–C)**. No correlations were found in control subjects **(D–F)**. *p < 0.05

**Figure 4:**
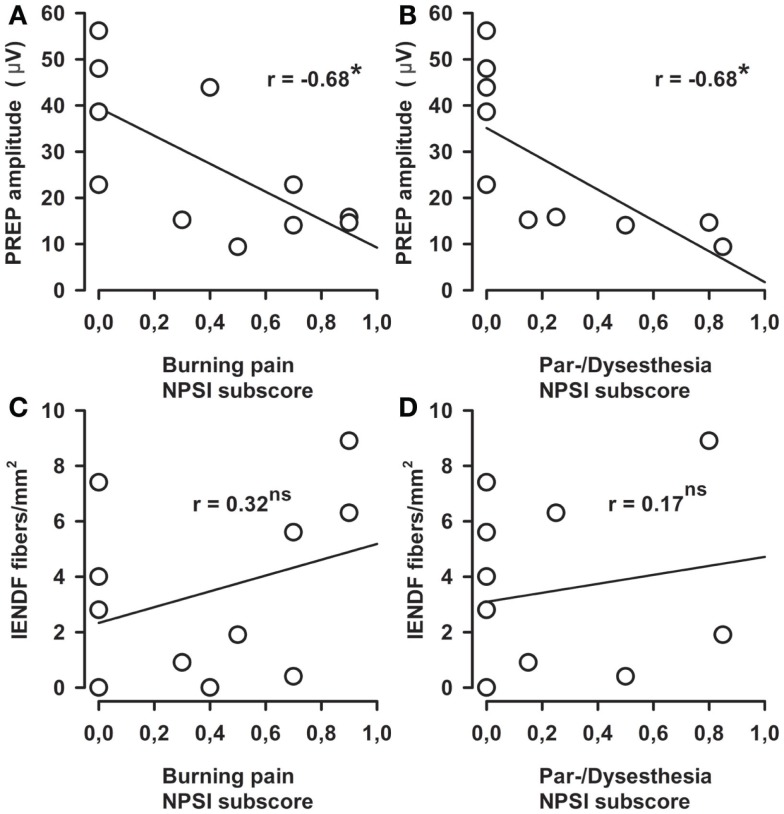
**Correlations between burning pain and abnormal sensation as well as pain-related evoked potential (PREP) amplitudes and intraepidermal nerve fiber density**. Negative correlation between PREP peak-to-peak amplitudes (PPA) and burning pain in the Neuropathic Pain Symptom Inventory (NPSI) subscore (*r* = 0.68) in mixed fiber neuropathy (MFN) patients is shown in **(A)**. Similar negative correlations appeared between PPA and abnormal sensations in the NPSI subscore [*r* = 0.68, **(B)**]. However, intra-epidermal nerve fiber density (IENFD) and burning pain in **(C)** and abnormal sensation in the NPSI subscore in **(D)** revealed no significant correlations. *p < 0.05

Stimulus intensities used to elicit PREP from the feet were not different between MFN patients and control subjects (Table 2). We analyzed pooled data (N1, P1, and PPA as well as stimulus intensities) from both sides of the lower legs since no side difference had been observed. N1 and P1 latencies as well as PPA amplitudes of PREP were not different between MFN and control subjects, except for a tendency toward prolonged N1/P1 latencies and reduced PREP amplitudes in MFN (Table 2; Figure 2 shows a representative example of a PREP trace of a control subject). Analysis of PREP parameters in the largest subgroup of CIDP patients (*n* = 6) compared to controls revealed no intergroup difference. Between PREP PPA induced at the feet and thermal perception thresholds (CDT, WDT, TSL) of MFN patients a positive correlation was encountered (Figures 3A–C). We noted a positive correlation for CDT in MFN patients (*r* = 0.71, *p* < 0.05, Figure 3A), as well as for WDT (*r* = 0.63, *p* < 0.05, Figure 3B) and TSL (*r* = 0.67, *p* < 0.05, Figure 3C). We found no correlation for CDT, WDT, and TSL with PREP PPA in control subjects (Figures 3D–F). CPT, and HPT *z*-scores as functions of small fibers and MDT, MPT, VDT, and PPT *z*-scores reflecting large fiber function did not correlate with PREP PPA in patients with MFN or in the controls. Moreover, we detected no correlations between PREP, sural nerve NCV, and sural nerve SNAP amplitude and controls. Burning pain quality and the presence of par- or dysesthesias assessed by the respective NPSI subscores correlated negatively with PREP PPA in MFN patients (*r* = −0.68, *p* < 0.05, Figures 4A,B). We observed no correlation between paroxysmal and evoked pain and PREP PPA in patients with MFN. Moreover, IENFD showed no correlation with the intensity of burning pain and the existence of par- or dysesthesias in the NPSI subscores of patients with MFN (Figures 4C,D). Pain intensity elicited by the electrical stimulus did not correlate with the PREP PPA in MFN patients and controls. Furthermore, the IENFD failed to correlate with either PREP latencies or amplitudes. In particular, we observed no correlation between the loss of IENFD in distal skin biopsies and burning pain or with par- and dysesthesias in MFN patients. In addition, we found no correlation between electrical stimulus intensity and IENFD.

**Table 2:** **Pain-related evoked potentials elicited at the foot and stimulus intensity data of patients and controls**.

Parameter	MFN patients	Controls	*p*-value
N1 latency (ms)	176 ± 47	162 ± 31	0.46
P1 latency (ms)	228 ± 44	210 ± 47	0.39
PPA (μV)	30 ± 15	39 ± 31	0.45
Current intensity (mA)	2.07 ± 0.37	1.8 ± 0.4	0.15

*ns, not significant; PPA, peak-to-peak amplitudes*.

Analyzing the NPSI subscores, burning pain was the most frequently reported pain quality in patients with MFN (64%), whereas less than half of the patients experienced pressure, evoked, or paroxysmal pain (36–47%; see Table S3A in Supplementary Material). In contrast, our healthy control subjects reported no burning, pressure, evoked or paroxysmal pain. In the McGill pain questionnaire, MFN patients characterized their pain using sensory and affective pain descriptors, whereas the controls reported no pain (see Table S3B in Supplementary Material). To define the predominance of Aδ- or C-fiber-mediated pain, the total amount of the “dull” or “pressing” pain descriptor selections was calculated from the McGill pain questionnaire data (23). This sum was higher than that of the selection “pricking” pain in 8/9 MFN patients with CIDP and vasculitic neuropathy who had completed the questionnaire correctly, indicative of C-fiber-mediated pain (2/11 patients refused to fill in the McGill questionnaire).

## Discussion

Our results demonstrate that, in addition to QST, PREP amplitudes may provide a useful parameter reflecting functional small fiber impairment in patients with painful MFN. This observation is underpinned by the strong correlation among increased thermal perception thresholds and reduced PREP amplitudes in patients with MFN, and by the correlation between ongoing burning pain as well as par- and dysesthesias and reduced PREP amplitudes.

Similar results have been obtained using other methods in small fiber neurophysiology. Nociceptive N2/P2 latencies were delayed and CHEP and LEP amplitudes reduced or even absent in MFN compared to healthy controls (7). The N2/P2 amplitude of LEP was reduced in patients with Charcot-Marie-Tooth disease type 1A (40). Two studies showed prolonged N1/P1 latencies and reduced PREP amplitudes in 13 patients with hepatitis C versus 28 healthy controls and nine patients with HIV-associated MFN compared to nine controls (11, 14). In our study, we detected neither prolonged N1/P1 latencies nor reduced PREP amplitudes in MFN patients compared to controls. This may be due to methodological differences (CHEP, LEP vs. PREP) and cohort size, and to our patient group’s heterogeneity.

Our MFN cohort consisted of different diagnostic subgroups (CIDP, vasculitic neuropathy, chronic axonal neuropathy). It is known that the type of neuropathy can affect cerebral pain-associated potentials; e.g., LEP may be absent in metabolic or toxic neuropathies (41). Thus, future large-scale studies enrolling patients from different MFN subgroups are necessary to determine whether the trend toward prolonged PREP latencies and reduced amplitudes is relevant in some types of MFN and masked by other subgroups of MFN patients.

Moreover, due to the small sample size of cohorts, demographic factors such as gender and age cannot be investigated by this study. It is of great interest to determine the influence of these factors on PREP parameters in a future large-scale study.

Due to the short anode-to-cathode distance design of the concentric electrode nociceptive afferents in the superficial skin are activated. By this electrode arrangement, a high current intensity is gained when choosing low current stimulation. Furthermore, the electrical stimulation caused a pin-prick sensation that is a phenomenon associated with the excitation of Aδ -fiber nociceptors (42–44), and the NCVs achieved by stimulating these concentric electrodes fall within the range of Aδ-fibers (10, 12, 45, 46). No PREP were detected at up to 2.5 mA of electrical stimulation intensity after topical application of lidocaine, which leads to a selective loss of thermal and pain sensation, but does not affect tactile sensation (47). These findings suggest that PREP are mediated by small-caliber nerve fibers (47). The response of the blink reflex was inhibited by 90% after topical application of lidocaine, indicating that the blink reflex response is contaminated by the co-activation of Aß-fibers by 10% (9). Co-activation of Aß-fibers can be induced by the concentric electrode (48, 49) indicated by latency gaps between PREP and LEP that are larger than the thermoreceptor activation time of 40 ms (48, 50). However, in this study, the amplitudes, morphology, and topographic localization were similar between PREP and LEP (48) suggesting that PREP amplitudes might be the more specific nociceptive marker than PREP latencies. The coactivation of Aß-fibers might be caused by employing a current intensity between 3 and 5 mA applied via the concentric electrode (49). Therefore, particular care must be taken to avoid additional Aß-fiber activation (e.g., by keeping stimulus intensities below 2.5 mA) during PREP measurements that are unnecessary when applying LEP (48). In our study, the electrical stimulation intensity was tuned below 2.5 mA, so that large and medium-sized fibers might have been unspecifically stimulated to a small but irrelevant extent.

Therefore, this factor probably does not account for the lack of a difference between our patients’ and controls’ PREP parameters. The characterization of late components of PREP needs future studies. This may help to differentiate between the excitation of different fiber types similar to the reported early and late components of LEP indicating Aδ- or C-fiber excitation (51).

Another issue is the potential influence of analgesic drugs on PREP parameters. As experimental *in vitro* evidence suggests, nociceptive transmission in the dorsal horn is altered by anticonvulsant drugs such as gabapentin and pregabalin (52, 53); human studies showed inhibitory effects of standard analgesic drugs on cortical pain potentials elicited by electrical stimulation (54). Thus, we cannot exclude a small inhibitory influence of long-term use of anticonvulsants with analgesic properties on PREP parameter in our study. However, we do not estimate a relevant effect affecting our results as these anticonvulsants are no direct analgesic drugs and do not block nociceptive transmission within the spinal cord. MFN can be characterized by QST in addition to examining nerve conduction and clinical symptoms. Increased mechanical (MDT), thermal (CDT, WDT), and vibration detection thresholds (VDT) imply the impairment of small and large nerve fibers (28, 29). The relationship between reduced PREP amplitudes and increased CDT (more pronounced than WDT and TSL) highlights PREP amplitudes as a function of Aδ- more than C-fibers. PREP amplitudes in MFN patients may thus be suitable for clinical application when pharmacotherapy or the MFN time course needs to be evaluated, although these correlations are not turning to account for a single patient. The relationship between PREP amplitudes and thermal perception is only detectable under pathological, not healthy conditions. This may indicate that the small fiber degeneration in skin alters both Aδ-mediated pain processing and thermal perception mediated by both Aδ- and C-fibers to a similar degree. Therefore, in pathological conditions like those that MFN patients experience, PREP are sensitive markers of small nerve fiber degeneration. Concordant with these findings, we did not detect any correlations between PREP amplitudes and QST parameters that reflect large fiber dysfunction such as VDT and MDT. Moreover, QST parameters that reveal C-fiber function such as HPT did not correlate with PREP amplitudes. This lack of correlation appears to reveal a potential role of PREP amplitudes in assessing primarily Aδ-fiber function. The correlation between PREP amplitudes and QST parameters reflecting small fiber dysfunction across different MFN subgroups in a small cohort supports PREP as a useful instrument for assessing small fiber dysfunction in addition to QST. Recording PREP at the feet is less time-consuming than the QST standard protocol, which would make easy administration in clinics potentially relevant in follow-up situations after pharmacotherapy or to evaluate therapeutic efficacy. Furthermore, as disturbances of thermal perception accessible by combining QST and PREP often precede large fiber dysfunction in polyneuropathies (55, 56), PREP and QST may make early diagnosis more feasible.

The absent correlation between skin biopsy results and PREP parameters is in agreement with antecedent reports (12, 13). This lack of correlation is most likely based on the fact that PREP predominantly measure Aδ fibers, whereas the intra-epidermal nerve fibers cover mostly C-fibers. In addition, PREP and skin biopsies were not obtained from exactly the same location (IENFD: lower leg, 10 cm upstream to the lateral malleolus; PREP: foot dorsum).

One reason why burning pain revealed no correlation with the IENFD in MFN patients might be that preserved C-nociceptors may cause spontaneous ectopic discharges resulting in pain (57–59). In a LEP study investigating patients with painful MFN of different origin, an inverse correlation was reported between LEP amplitudes and spontaneous burning pain, while evoked pain did not correlate with LEP (60). Damage to nociceptive fibers that have lost their intra-epidermal terminals was assumed to be the underlying pathophysiological mechanism for the latter (60), which may also apply to our findings.

Different pain phenomena might indicate potential pain mechanisms in MFN patients (60, 61). This opens the avenue to improved therapeutic strategies based on mechanisms instead of etiologies. Our cohort tended to complain of burning pain supposedly mediated via C-fibers (62). This finding concurs with the predominance of C-fiber versus Aδ-fiber-mediated pain in our findings from the McGill pain questionnaire in CIDP and vasculitic neuropathy patients (23). It is not surprising that C-fiber-mediated pain dominates pain sensation in neuropathic pain states, as C-nociceptors demonstrate high spontaneous activity in painful neuropathies, as microneurography demonstrates (59). Furthermore, experimental rat models have shown that abnormal peripheral C-nociceptor discharges with multiple spiking due to altered conduction in nociceptors may account for neuropathic pain (57). In the MFN Guillain-Barré syndrome, IENFD was lower in patients without pain than in those suffering from neuropathic pain (17). This finding supports the notion that preserved C-fibers are major contributors to neuropathic pain in MFN.

## Conclusion

In summary, our data suggest that PREP amplitudes may be a sensitive and non-invasive marker of small fiber impairment in painful MFN in conjunction with QST. However, it is crucial to endorse that the clinical diagnosis of small fiber impairment in MFN can only be made by performing other small fiber tests in addition to recording PREP. Our findings support PREP amplitudes as an adequate tool to measure small fiber conductivity in patients with MFN or for follow-up after pharmacotherapy. Large-scale future investigations are required to demonstrate the utility of PREP in different subgroups of MFN patients as an instrument for better differential diagnosis and treatment.

## Author Contributions

NH, NÜ, CS wrote the manuscript, designed the study, and undertook statistical analysis; NH and AK performed the experiments; CS, DZ, NÜ, NH analyzed data; ZK revised the manuscript for relevant intellectual content.

## Conflict of Interest Statement

The authors declare that the research was conducted in the absence of any commercial or financial relationships that could be construed as a potential conflict of interest.
