# Supplementary material for: Amplitudes of Pain-Related Evoked Potentials Are Useful to Detect Small Fiber Involvement in Painful Mixed Fiber Neuropathies in Addition to Quantitative Sensory Testing – An Electrophysiological Study
Source: Front Neurol. 2015 Dec 7;6:244. doi: 10.3389/fneur.2015.00244 (PMC4670913; doi:10.3389/fneur.2015.00244)
Supplement: Supplementary file 1 [file Table_1.DOCX]

**Supplementary data:**

**Table 1: Clinical characteristics and distal intraepidermal nerve fiber density of mixed fiber neuropathy patients**

**Patient Diagnosis Hypoesthesia Thermhypo- Pallhypo- Paresis Hypo- IENFD**

**esthesia esthesia reflexia fibers/mm**

1 sensory CIDP + + + - + 0.9

2 CIDP + + + + - 0

3 axonal sensory-motor neuropathy + + + - + 2.8

of unknown etiology

4 non-systemic vasculitic neuropathy + + + + + 4

5 CIDP + - + + + 5.6

6 CIDP with monoclonal + - + + + 6.3

gammopathy

7 vasculitic neuropathy in CREST syndrome + + + + + 1.9

8 vasculitic neuropathy in + - + + + 7.4

Churg Strauss syndrome

9 sensory CIDP + - + - + 0

10 axonal sensory-motor neuropathy of unknown + - + - + 0.4

etiology

11 CIDP + + + + + 8.9

Abbreviations: CIDP = chronic inflammatory demyelinating polyneuropathy; CREST syndrome = Calcinosis, Raynaud`s Syndrome, Esophageal dysmotility, Sclerodactyly, and Telangiectasia syndrome; IENFD = intraepidermal nerve fiber density.

+ = symptom is present, - = symptom is absent.
