# Supplementary material for: Amplitudes of Pain-Related Evoked Potentials Are Useful to Detect Small Fiber Involvement in Painful Mixed Fiber Neuropathies in Addition to Quantitative Sensory Testing – An Electrophysiological Study
Source: Front Neurol. 2015 Dec 7;6:244. doi: 10.3389/fneur.2015.00244 (PMC4670913; doi:10.3389/fneur.2015.00244)
Supplement: Supplementary file 2 [file Table_2.DOCX]

**Supplementary data:**

**Table 2: QST raw data**

**Patients Controls**

CDT -10.6 ±6.4 °C -2.6 ± 2.6 °C

WDT 11.3 ± 4.3 °C 6.1 ± 3.2 °C

TSL 29.1 ± 6.5 °C 13.6 ± 6.7 °C

CPT 10 ± 0 °C 13.5 ± 5.4 °C

HPT 48.6 ± 1.8 °C 45.1 ± 3.1 °C

MDT 66.9 ± 157.8 mN 2.0 ± 1.4 mN

MPT 102.2 ± 96 mN 30.1 ± 1.9 mN

MPS 3.2 ± 5.5 pain rating (NRS 0-100) 5 ± 8.4 pain rating (NRS 0-100)

VDT 3.4± 2.4 /8 7.2 ± 0.7 /8

PPT 568.3 ± 142 kPa 515 ± 99 kPa

**Abbreviations:** °C = temperature, degree in Celsius, CDT = cold detection threshold, CPT = cold pain threshold, HPT = heat pain threshold, kPa = kilo Pascal, MDT = mechanical detection threshold, MPS = mechanical pain sensitivity, MPT = mechanical pain threshold, mN = milli Newton, NRS = numeric rating scale, PPT = pressure pain threshold, TSL = thermal sensory limen (ability to detect temperature changes), VDT = vibration detection threshold, WDT = warm detection threshold (WDT).
