# Supplementary material for: Amplitudes of Pain-Related Evoked Potentials Are Useful to Detect Small Fiber Involvement in Painful Mixed Fiber Neuropathies in Addition to Quantitative Sensory Testing – An Electrophysiological Study
Source: Front Neurol. 2015 Dec 7;6:244. doi: 10.3389/fneur.2015.00244 (PMC4670913; doi:10.3389/fneur.2015.00244)
Supplement: Supplementary file 3 [file Table_3.DOCX]

**Supplementary data:**

**Table 3: Pain profile of the mixed fiber neuropathy patients**

1. **NPSI subscores**

**Patients Controls p-value**

**Mean ± SD in % vs. controls**

Burning pain 0.4 ±0.35 64% 0 p<0.05

Pressure pain 0.32 ±0.34 47% 0 p<0.05

Paroxysmal pain 0.22 ±0.31 36% 0 p<0.05

Evoked pain 0.13 ±0.23 47% 0 p<0.05

Abnormal sensation 0.23 ±0.32 45% 0 p<0.05

1. **McGill pain questionnaire: significant items vs. controls, p<0.05**

**Classes Pain descriptors Patients Controls**

Spatial Flashing 1.22±1.54 0

Constrictive pressure Cramping 2±1.88 0

Thermal Burning 1.33±1.69 0

Dullness Aching 2.22±1.82 0

Heavy 1.77±1.82 0

Tension Tiring 0.77±1.03 0

Punishment Punishing 1.55±1.64 0

Evaluative Annoying 2.11±1.79 0

Sensory: miscellaneous Spreading 1± 1.24 0

Numbing 2±1.88 0

Drawing 1.8±1.91 0

Squeezing 1.33±1.63 0

Sensory Cold 0.89±1.28 0

Affective-evaluative: Nagging 1.55±1.83 0

Miscellaneous Agonizing 1.22± 1.61 0

Abbreviations: NPSI = Neuropathic Pain Symptom Inventory, SD = standard deviation.
